# What Predicts Patients’ Willingness to Undergo Online Treatment and Pay for Online Treatment? Results from a Web-Based Survey to Investigate the Changing Patient-Physician Relationship

**DOI:** 10.2196/jmir.5244

**Published:** 2016-02-04

**Authors:** Johanna Roettl, Sonja Bidmon, Ralf Terlutter

**Affiliations:** ^1^ Alpen-Adria Universitaet Klagenfurt Department of Marketing and International Management Klagenfurt Austria

**Keywords:** physician-patient relationship, online treatment, general practitioners, willingness to pay

## Abstract

**Background:**

Substantial research has focused on patients’ health information–seeking behavior on the Internet, but little is known about the variables that may predict patients’ willingness to undergo online treatment and willingness to pay additionally for online treatment.

**Objective:**

This study analyzed sociodemographic variables, psychosocial variables, and variables of Internet usage to predict willingness to undergo online treatment and willingness to pay additionally for online treatment offered by the general practitioner (GP).

**Methods:**

An online survey of 1006 randomly selected German patients was conducted. The sample was drawn from an e-panel maintained by GfK HealthCare. Missing values were imputed; 958 usable questionnaires were analyzed. Variables with multi-item measurement were factor analyzed. Willingness to undergo online treatment and willingness to pay additionally for online treatment offered by the GP were predicted using 2 multiple regression models.

**Results:**

Exploratory factor analyses revealed that the disposition of patients’ personality to engage in information-searching behavior on the Internet was unidimensional. Exploratory factor analysis with the variables measuring the motives for Internet usage led to 2 separate factors: perceived usefulness (PU) of the Internet for health-related information searching and social motives for information searching on the Internet. Sociodemographic variables did not serve as significant predictors for willingness to undergo online treatment offered by the GP, whereas PU (B=.092, *P*=.08), willingness to communicate with the GP more often in the future (B=.495, *P*<.001), health-related information–seeking personality (B=.369, *P*<.001), actual use of online communication with the GP (B=.198, *P*<.001), and social motive (B=.178, *P*=.002) were significant predictors. Age, gender, satisfaction with the GP, social motive, and trust in the GP had no significant impact on the willingness to pay additionally for online treatment, but it was predicted by health-related information–seeking personality (B=.127, *P*=.07), PU (B=–.098, *P*=.09), willingness to undergo online treatment (B=.391, *P*<.001), actual use of online communication with the GP (B=.192, *P*=.001), highest education level (B=.178, *P*<.001), monthly household net income (B=.115, *P*=.01), and willingness to communicate with the GP online more often in the future (B=.076, *P*=.03).

**Conclusions:**

Age, gender, and trust in the GP were not significant predictors for either willingness to undergo online treatment or to pay additionally for online treatment. Willingness to undergo online treatment was partly determined by the actual use of online communication with the GP, willingness to communicate online with the GP, health information–seeking personality, and social motivation for such behavior. Willingness to pay extra for online treatment was influenced by the monthly household net income category and education level. The results of this study are useful for online health care providers and physicians who are considering offering online treatments as a viable number of patients would appreciate the possibility of undergoing an online treatment offered by their GP.

## Introduction

### The Changing Patient-Physician Relationship

The relationship between a physician and a patient is a very delicate conjunction. When selecting a general practitioner (GP), patients take multiple factors into consideration in addition to their location or office hours. Other factors, such as the ability to communicate, to develop trust, and the engagement of the physician regarding the patient’s care, are also important factors in the selection process [1]. When patients feel well informed, they are more likely to follow the medication and treatment plan prescribed by a physician [2]. Within the last few years, there has been an enormous increase in demand for physicians’ time (eg, for consultations and treatments). This can be explained by the increasing need for primary health care management of chronic diseases and preventive medicine [3]. The patient’s role in the medical decision-making process has also shifted over the past few years. The relationship is changing from one in which patients follow the physicians’ orders to one in which decisions are made together, between the physician and the patient, and this consensual decision-making process requires more time than a top-down process. Patients are empowered to change their behavior to achieve better health care results [1,4-6]. Emanuel and Emanuel [5] outline 4 models to describe the increasingly complex patient-physician relationship, which is often characterized by conflicts between health and autonomy and conflicts between differing values held by the physician and patient. The models emphasize differing opinions about the goals of the interaction of physician and patient, of the physician’s obligations in the relationship, of the role that the patient’s values play, as well as of the level of patient autonomy.

According to the paternalistic model, shared objective criteria exist regarding what is best for the patient’s well-being. These criteria are known to the physician and the physician decides which interventions would be appropriate. These interventions might even sometimes be contradictory to the patient’s own opinion. The physician has the role of a guardian and provides the patient with little information. Possible scenarios where paternalistic care proves necessary might include, for example, cases involving acute or trauma care when treatment is needed immediately. The second model is the informative model, which assumes that the physician provides relevant factual information about available treatment possibilities to the patient, but it is the patient who selects the medical treatments based on his/her values. The physician then implements the patient’s selection of intervention. This model comprises increased involvement and high autonomy of the patient, with the decision making being a shared effort. The physician’s role is that of a competent technical expert. The third model is the interpretive model, where the physician’s obligation is not only to provide the patient with factual information, but the physician also explains and interprets the patient’s values. This model assumes that the patient’s values are often not fixed and that they sometimes might even be unknown to the patient. The physician elucidates the patient in understanding the values and informs the patient about possible interventions, but the patient makes the ultimate decision. The physician behaves as an adviser or counselor to the patient. The fourth model is the deliberative model, which assumes that the physician should help the patient to reflect his/her preferences and health-related values before making a decision. The patient’s autonomy is high and the patient’s values are open to development and revision through moral discussion. The physician’s obligation is to articulate and persuade the patient of the most admirable values as well as to inform the patient and implement the patient’s selected intervention. The physician’s role is characterized as that of a friend or a teacher [5].

The Internet is gaining increasing importance in the patient-physician relationship and is relevant to each of the 4 models. The patient-physician relationship via the Internet can range from simple information provision and searching (eg, the physician puts office hours and addresses on the website and the patient looks them up) to more sufficient interactions (eg, they both exchange documents via email or the patient rates a physician on a physician-rating website) to very complex interactions, such as the substitution of a personal face-to-face meeting in the physician’s office by a virtual meeting on the Internet. For instance, advice from a physician to a patient regarding possible medication options via a simple email might be found in the paternalistic or informative model, whereas a more intensive online video meeting might be found in the interpretive or deliberative model. Patients and physicians have to adapt to this new form of health care [7,8].

With regard to Europe, empirical evidence regarding the application of the 4 models developed by Emanuel and Emanuel is scarce. However, the study by Falkum and Førde [9] conducted among physicians in Norway found similar dimensions as those described by Emanuel and Emanuel [5]. Falkum and Førde detected 3 dimensions of the patient-physician relationship: paternalism, patient autonomy, and moral deliberation. Based on a survey conducted with 990 physicians, the results indicated that the physicians’ gender, country of graduation, practice type, personal illness experience, or workplace were not statistically significant in association with any of the 3 dimensions, whereas the age of the physicians and their specialty were related to paternalism. All respondents agreed that patient information and patient consent should be the central focus in modern medical treatments. More than 80% of the respondents reported that the patient is a “customer,” who should be informed about different treatment alternatives by their physician. Almost half of the physicians stated that the physician is an expert and, therefore, should decide what he/she thinks would be the best for the patient under most medical circumstances. And 40% of the respondents indicated that it is a burden for the doctor most of the time when the patient is involved in the treatment decision because the patient often lacks relevant medical knowledge. In addition, almost 80% of the respondents agreed the patients should have the right to choose the treatment that matches his or her own values most closely. From this study, it can be argued that the physicians’ attitudes toward paternalism, the patients’ autonomy, and the moral deliberation is quite ambiguous. The doctor’s empathetic understanding of each single patient should be at the very center of the patient-physician communication [9].

### Patients’ Use of the Internet for Health-Related Information Seeking

The use of the Internet as a source of health information by patients has increased rapidly in many Western societies within the last few years [10,11]. An increasing number of people want to gain a more collaborative view of their own health and use the Internet as an aid to self-diagnosis and self-medication, which leads to the “empowered patient” [2]. In the past, the physician typically held the majority of the information and power and provided the patient with selected information. Now, because patients have access to an enormous quantity of health-related information through the Internet [11], the asymmetry of information in the patient-physician relationship is decreasing. A national survey conducted by the Pew Internet & American Life Project in 2013 showed that 72% of US adults who use the Internet have searched online for health-related information (representing 59% of all US adults). More than one-quarter (28%) base their decision about whether or not to visit a physician on online health-related information. Most US adults (70%) use the Internet primarily to obtain health-related information to inform themselves and/or to change their decision about a treatment for their illness, whereas half of US adults (50%) use the Internet to find answers to specific health-related questions or to get different opinions from other physicians or Internet users. From a demographic point of view, women are more prone to searching for health-related information than men, and younger people use the Internet to obtain health-related information more often than older individuals do. In the United States, Internet users between the ages of 30 and 64 years are the most likely group to consult or post online reviews and rankings of health treatments and services. Furthermore, Internet users with a higher level of education are more likely to consult or post online health-related reviews and rankings in comparison to those with a lower level of education. The same is true for people with a higher annual household income compared to those with a lower annual household income [12].

### Online Treatments

Online treatments are gaining popularity with more and more patients using online health care providers for personal health care issues and GPs consulting and advising the patients by telephone, email, videoconferences (eg, Skype), or online [10,13]. The treatments can be differentiated into online treatments offered by the patients’ local and personally known GPs, and online treatments offered by unknown GPs associated with an already existing online health care provider (eg, DoctorSpring.com, MeMD, or Teladoc) in countries where the health care system offers this possibility by law. In place of personal face-to-face appointments, patients can be treated online by GPs, which means that a consultation or a treatment can be offered to patients without requiring their physical presence in the physician’s office [3,14]. The patients can communicate about nonemergency health care issues online with the GPs from home, work, or any place equipped with Internet access [15]. For face-to-face treatments, the patients only need a computer or mobile device (eg, mobile phone or tablet) with Internet access, a video camera, and a microphone, which are integrated in most modern devices anyway [3,15,16]. If the patients wish to undergo an online treatment with an unknown GP of an online service provider, an account has to be created. Afterwards, data specification is often needed about the patients’ health record, lifestyle, family history, as well as information about their usual GP and pharmacy. Payment information should also be determined; in a next step, individual appointments can be made (eg, during the lunch break, late at night after work, or on weekends) to discuss personal health-related issues at the earliest opportunity [14,17-19].

Many different types of online consultation exist, some for acute conditions, such as minor infections, and others for the management of chronic conditions or for consulting with patients with nonurgent acute health care concerns, such as a needed prescription (ie, for colds, flu, allergies, urinary tract infections, or acne), which should be sent to the patient’s pharmacy, or for laboratory tests which should be ordered [3,14,20].

In an established online medical treatment, the patient reports his or her symptoms in a standardized way. The GP reviews the symptoms and diagnosis or treatment plans are made. This can include prescriptions for medicines or advice regarding follow-up care [15]. Online treatments might be relevant for each of the 4 models of the physician-patient relationship developed by Emanuel and Emanuel [5]. Communication between the patient and the physician about factual information can be easily conducted via email. For instance, if the physician knows the patient personally and the patient needs medical treatment or advice (eg, needs a prescription for medicine for a chronic disease), information through email communication can be shared. This could be the case in the paternalistic or in the informative model. Or when deciding about different treatment options (eg, different possible therapies), an online video consultation might be used. The online video meeting of physician and patient offers similar possibilities to discuss treatments and options or to reflect on the patient’s values and preferences. Interventions can also be discussed and selected jointly in an online video meeting, as would be the case in the interpretive model or in the deliberative model.

#### Advantages of Online Treatments

From the patients’ point of view, online treatments may offer several advantages when requiring an online consultation from a GP, such as no waiting time for an appointment, no waiting time in the doctor’s waiting room, and no traveling to the physicians’ office, which saves money and time in the long run [3,15,16]. Furthermore, if patients are timid or easily intimidated when communicating face-to-face with their physician, online interactions may make them feel more at ease, given that the interaction is mediated by technology. They may even elect to remain anonymous. It might well be that patients who undergo online treatments may sometimes be less shamefaced and may talk more openly, which might be especially relevant in the context of personal health care issues that are embarrassing for patients [21-23]. Additionally, many physicians working for online health care providers cooperate with online pharmacies, where the prescribed medicine can be ordered directly and is subsequently sent by post without delay to the patient’s private address (eg, DrEd.com) [24]. Another advantage of using an online treatment offered by a GP from an online health care provider is that the patients can contact the GPs 24/7. For physicians, online treatments offer the possibility to optimize their productivity, improve chronic disease management, and control their time schedule better, such as by filling a patient’s cancelation with an e-visit or by working more flexibly in the evenings, on the weekends, or from home [7,25-27]. In addition to the already mentioned positive aspects of online treatments, lower costs compared to traditional office settings of GPs are also advantageous [13,20].

#### Disadvantages of Online Treatments

Despite the clear advantages of online treatments, adverse aspects also exist, including concerns about the quality of online treatments; for example, whether GPs can make a precise diagnosis without seeing the patient face-to-face and performing a medical assessment in form of a real physical examination. Additionally, there are also concerns about appropriate follow-up visits and accurate prescriptions of medication [3,14,28]. Other obstacles regarding online treatments are concerns about the patients’ privacy and sensitivity of information. The adoption of online treatments has been slow because of the complexity of effective electronic communication, difficulties in reimbursement, and privacy concerns [15,29]. From the patients’ point of view, establishing contact with an unknown GP through a service provider bears the risk of being unable to judge the trustworthiness and expertise of the GP. On the other hand, physicians often fear being inundated with online messages from the patients, which they cannot answer in detail in a timely manner [30,31].

#### Willingness to Undergo Online Treatments

As far as we know, European studies until now have only examined health-related patient-physician communication and the willingness to undergo an online consultation by email, whereas recently conducted studies in the United States have already investigated patients’ willingness to undergo online treatments through video.

In 2007, 7.4% of 1021 Danish respondents of a national survey conducted in 7 European countries (N=7022) reported having contacted a family doctor, specialist, or other health professionals via email or the Internet to request or renew a prescription. In all, 9.9% of respondents reported having scheduled an appointment and 6.7% had asked specific health-related questions via email or the Internet. In comparison, respondents from Portugal indicated no email usage for health communication. In general, Danes reported the highest willingness to undergo an online consultation with their GP (26.2%) [8]. Another survey canvassing 14,000 citizens across 14 European countries in 2011 found that more than a quarter of all participants reported sending or receiving emails from their doctors, nurses, or health care organizations. Statistically significant differences among countries were found; Denmark reported the highest level of sending/receiving emails (50.7%) and participants from France reported the lowest level (18.7%). Respondents from Denmark, Estonia, Italy, and Sweden were more willing to use email for health-related communication in comparison to those from France, Belgium, Slovakia, Slovenia, and the United Kingdom [32]. The high reported level of email communication in Denmark is in accordance with the Danish compulsory primary care services for physicians to offer email contact and online services to their patients [33,34]. Furthermore, more men than women, younger respondents aged between 16 and 24 years, and people with higher education used email for health care communication [32].

According to a December 2014 online survey by The Harris Poll of 2019 US adults aged 18 years and older, almost 64% of respondents were willing to see a doctor online using video. Of those who were willing to consult their doctor over video, 61% listed convenience as the main determinant for their willingness. The survey also showed that 11% of patients aged 18 to 34 years, 8% of patients aged 35 to 44 years, 5% of patients aged 45 to 64 years, and 4% of patients aged 65 years and older would switch to an online visit with a GP, indicating that willingness decreases with age. Furthermore, a majority of the respondents (70%) would prefer to receive a prescription after an online video visit with the physician if medication is necessary [35]. Another survey revealed that only 11% of US households with broadband Internet access prefer an online video consultation with their physician compared to almost 70% who prefer in-person visits conducted at their physician’s office. The most likely patient segments to use online health care communication tools have a mean household income of US $50,000 or more, which is approximately the median household income in the United States [36]. According to a report by Parks Associates [37], it is expected that by 2018 more than 65% of US households with an Internet connection will use virtual health care video consultations with a GP.

#### Willingness to Pay for Online Treatments

According to a 2006 study in the United States by Adler [30], who endeavored to evaluate current patient readiness and willingness to pay for online services, more than three-quarters (n=185) of all interviewed patients with Internet access were willing to pay a small annual fee for online services, including appointment requests, billing inquiries, medication prescription refills, having email contact with their GP, and viewing parts of their medical record. Willingness to pay did not significantly vary by age. Furthermore, the study showed that the most important online services for patients with Internet access (n=248) were conducting email correspondence with their physician (34%), viewing parts of their medical record online (22%), and refilling prescriptions for medication (11%) [30]. In comparison, a more current study from 2013, conducted by the Pew Internet & American Life Project, showed that 26% of US Internet users who have searched online for health-related information have already been asked to pay a certain amount to gain access to health-related information. However, only 2% of those asked to pay actually did so [12]. According to the results of the previously mentioned study, conducted by American Well, 62% of respondents were of the opinion that online treatments should cost less than in-person visits [35].

### Technology Acceptance Model in the Patient-Physician Relationship

The Technology Acceptance Model (TAM) is a model that describes and predicts the acceptance and use of new information technologies and is applied in different contexts of online consumer behavior and online health information [38,39]. According to the model, different attributes influence the users’ decisions about their acceptance of the technology. The model comprises 2 central beliefs about a new technology—the perceived usefulness (PU) and the perceived ease-of-use (PEOU)—which influence the behavioral intention to adopt a certain technology [40-46]. By definition, PU is “the degree to which a person believes that using a particular system would improve his/her performance” [47]. The PEOU is defined as “the degree to which a person believes that using a particular system would be free of effort” [47]. In our study, we define PU as “the usefulness of the Internet to gain health-related information” and PEOU as “the perceived ease-of-use of the Internet to gain health-related information” [38].

Although studies have shown that PU and PEOU influence the behavioral intention to use health information technologies (eg, the Internet) positively [48-51], and given the expanding role of the Internet regarding health-related information in the patient-physician relationship, there has been little discussion about what kind of variables may predict willingness to undergo online treatment and willingness to pay additionally for online treatment offered by the GP. Therefore, this study analyzes several variables to predict willingness to undergo online treatment and willingness to pay for online treatment offered by the GP.

This study’s purpose is to address the following objectives:

1. Identify sociodemographic and psychosocial variables as well as variables of Internet usage that predict willingness to undergo online treatment.
2. Identify sociodemographic and psychosocial variables as well as variables of Internet usage that predict willingness to pay additionally for online treatment offered by the GP.

Sociodemographic variables include age, gender, highest education level, and monthly household income. Psychosocial variables contain the constructs of health-related information–seeking personality, social motive, and trust in the GP. Variables of Internet usage refers to a set of variables, which are termed as actual use of online communication with the GP, perceived usefulness of the Internet for health-related information (PU), willingness to communicate online with the GP more often in the future, and willingness to undergo online treatment offered by the GP.

## Methods

### Participant Recruitment

An online survey of 1006 randomly selected German patients was conducted in September 2012. The sample was drawn from an e-panel maintained by GfK HealthCare (Gesellschaft für Konsumforschung), a leading survey research company in Nuremberg, Germany. The term “patients” in this study refers to individuals who have visited a physician at least once in the previous 3 months before the beginning of the study. In total, 20 respondents were excluded from the analysis because of inconsistent answer patterns (eg, flatliners or contradictions) or an extremely short answer time. Another 28 participants were excluded from the study because their number of missing values exceeded the limit of 30% [52]. Missing values were imputed with SPSS version 22 (IBM Corporation, Armonk, NY, USA). In total, 958 usable questionnaires were analyzed by using 2 multiple regression models. Small amounts of money were offered as incentives to participate in the survey and fill out the questionnaire.

### Questionnaire

The questionnaire was designed by the researchers based on the existing literature. Originally, the online questionnaire was in German. Available scales from the literature were used where applicable. The literature used for the scales is quoted in square brackets within the text as well as in Multimedia Appendix 1, where an excerpt of the questionnaire can be perused. Items for which no literature are quoted were developed by the researchers. All items were measured by 7-point rating scales, except the categorical variables. As an alternative, all items had a “no answer” category. The denotation of the items (D1 to D8, F11_1 to F34) in parentheses refers to Multimedia Appendix 1.

### Measurement of Sociodemographic Variables

Age (D2_1) was measured by asking the patient’s year of birth. Gender (D1) was measured by single items (1=male, 2=female). The highest education level was measured through the inquiry about the participant’s highest completed level of education (D4). An indication of the monthly household income was also requested (D8).

### Measurement of Psychosocial Variables

#### Health-Related Information–Seeking Personality

Health-related information-seeking personality refers to the phenomenon that some patients have a higher need for cognition and information than others when making decisions as a patient. The need for cognition is a tendency of engagement as well as enjoyment in cognitive efforts, which means that people with a high need for cognition are more willing to engage in information-seeking activities in comparison to people with a lower need for cognition, who are less willing to do so. Furthermore, people who are more prone to seeking information are more likely to evaluate the information thoroughly, use more information sources, and rely more on the information [53,54]. Hence, patients with high levels of health-related information-seeking personality tend to inform themselves extensively when visiting a physician by searching for health-related information [55]. The health-related information-seeking personality scale (F20) consists of 9 items, developed by the researchers, partially adapted from the health information orientation scale derived by Dutta-Bergman [56] as well as by Simon et al [57] and Wilson and Lankton [58].

#### Social Motive

Patients’ motives for using the Internet for health-related information searches were measured based on 18 items partly derived from literature; some items were added by the researchers. The possibility to access different Web portals (eg, social networks, podcasts, or health forums) (F11_9) [7,59,60], the social component of establishing contact with someone easily (F11_11) [7,61], the need to be up-to-date (F11_12) [7], the preference for gathering information anonymously (F11_13) [7,60], and the possibility of sharing knowledge with others (F11_15) [7] were measured by multi-item scales. Items measuring fun (F11_17) and entertainment (F11_18) were adapted from Shih [62], Davis et al [63], and Venkatesh et al [44,64].

#### Trust in the General Practitioner

The respondents’ trust in their GP (F34) was examined by asking the following question: “How much do you trust your GP?” (1=no trust at all, 7=very high trust).

### Measurement of Variables of Internet Usage

#### Actual Use of Online Communication With the General Practitioner

To assess the respondents’ actual use of online communication with their GP (F13), respondents were asked to indicate how often they use the Internet to communicate with their GP. The answer scale ranged from daily, weekly, less frequently than once per week, monthly, less frequently than once per month, to never. The item was measured on a 6-point ordinal scale, reverse-coded, with a lower frequency revealing a higher score of actual use. This item was recoded for analyses for better readability.

#### Perceived Usefulness of the Internet for Health-Related Information

Perceived usefulness of the Internet to gain health-related information (F11_1 to F11_5, F11_14) was measured by existing multi-item scales partly derived and adapted from Venkatesh and Davis [40,44,47] as well as from other relevant literature [7,45,59,60].

#### Willingness to Communicate Online With the General Practitioner More Often in the Future

The willingness to communicate online with the GP more often in the future (F15) was measured by asking the respondents the following question: “Can you imagine using the Internet more often in the future for communication with your GP?” (1=highly unlikely, 7=very likely).

### Measurement of Willingness to Undergo Online Treatment Offered by the General Practitioner

To measure the willingness to undergo an online treatment offered by the GP (F18), respondents were asked to indicate the importance of being able to undergo online treatment by the GP (1=not important at all, 7=very important).

### Measurement of Willingness to Pay Additionally for Online Treatment Offered by the General Practitioner

Willingness to pay additionally for an online treatment offered by the GP was measured by asking the following question: “Indicate how willing you would be to pay a certain amount additionally for online treatment.” Participants could indicate their agreement on a scale ranging from 1 (“I would not be willing at all”) to 7 (“I would be willing”) (F19).

## Results

### Sample Characteristics

In total, 54.0% (517/958) of the participants were male and 46.0% (441/958) were female. The mean age was 43.73 (SD 13.00) years and 57.0% (546/958) of the respondents had a higher level of education (high school diploma or higher).

Regarding the variable gender, the data for this sample represent the German online population quite well compared to German Internet users in 2012 [65] (Table 1). With reference to the variable age, participants in our study were slightly older than the German Internet population. However, participants in our study had a minimum age of 18 years and the minimum age in the dataset used by the German Internet users was 10 years. With regard to the variable education, participants in our study were more highly educated than the German online population [65]. No comparable data could be found referring to the variable monthly household net income because the Federal Statistical Office (Statistisches Bundesamt) does not provide this information in their German Internet population dataset.

**Table 1:** Sociodemographic data of the sample in comparison with the German Internet population (2012).

Variable and categories		Total N=958	German Internet users^a^ N=58,556,000
**Gender, n (%)**			
	Male	517 (54.0)	29,553,000 (51.81)
	Female	441 (46.0)	27,492,000 (48.20)
Age (years; range 18-70 years), mean (SD)		43.73 (13.0)	>10
**Age categories (years), n (%)**			
	<44 years	471 (49.2)	32,896,000 (57.60)
	45-70 years	487 (50.8)	24,147,000 (42.34)
**Education, n (%)** ^b^			52,589,000^b^
	Without school qualification	4 (0.4)	
	Secondary general school	13 (1.4)	9,487,000 (18.04)^c^
	Polytechnic secondary school	120 (12.5)	
	Intermediate secondary school	269 (28.1)	29,467,000 (56.03)^d^
	Matura examination or higher	545 (57.0)	13,635,000 (25.93)^e^
**Monthly household net income (€), n (%)**		347 (100)	
	<1500	77 (22.2)	
	1500-2500	97 (28.0)	
	2501-3500	94 (27.1)	
	3501-4500	53 (15.3)	
	>4500	26 (7.5)	

^a^ Rounded to 1000 people. Projected number of Germans who used the Internet in the last 3 months. Age limit for questions concerning education and occupation: 16 years.

^b^ For the German Internet users, low education corresponded with levels 0, 1, and 2 of the ISCED classification system (up to secondary general school), medium education corresponded with levels 3 and 4 of the ISCED classification system (up to university entrance qualification), and high education corresponded with levels 5 and 6 of the ISCED classification system (higher than matura examination respectively university entrance qualification).

^c^ Low education.

^d^ Medium education.

^e^ High education.

### Exploratory Factor Analyses

The facets of patients’ personalities to engage in information-searching behavior on the Internet (9 items) were analyzed by an exploratory factor analysis (EFA) leading to a single-factor solution explaining 52.64% of variance, reflecting the personal tendency of information-searching behavior on the Internet. The factor was labeled “health-related information–seeking personality” and the factor scores were saved and used for the multiple regressions. The second EFA was executed with the variables measuring the motives for Internet usage (18 items), leading to 2 separate factors: PU of the Internet for health-related information searching and social motives for information searching on the Internet, explaining 63.74% of variance. Items with loadings below 0.45 or with loadings on both of the factors were eliminated. The construct of PEOU did not turn out to be a distinct factor according to the EFA for the remaining motivational items. Thus, in reference to the Eigenwert criterion, only 2 factor scores (PU and social motive) reflecting the contents of the remaining motivational items were saved for each respondent as variables for the following multiple regressions.

### Multiple Regression Analyses

Before performing the multiple regression analyses, we calculated the means and standard deviations of the dependent variables “willingness to undergo online treatment offered by the GP” and “willingness to pay additionally for online treatment offered by the GP” to estimate the overall willingness to undergo online treatments and pay for them. The frequency distribution of the 2 dependent variables is shown in Multimedia Appendix 2. The mean of the variable “willingness to undergo online treatment offered by the GP” was 3.60 (SD 2.02) on a 7-point scale; hence, it was slightly below the midpoint of the scale. By adding the percentages of those who marked the highest and lowest 2 points of the willingness to undergo online treatment answer scale, we found out that 19.9% (191/958) of the respondents indicated a high willingness to undergo online treatment in comparison to 36.5% (350/958) who reported a low willingness. The mean of the variable “willingness to pay additionally for online treatment offered by the GP” was a low 2.30 (SD 1.81) on a 7-point scale. Among those who marked one of the 2 highest points of the willingness to pay additionally answer scale, less than 10% (8.7%, 83/958) were willing to pay for an online treatment additionally.

#### Multiple Regression Analysis to Predict Willingness to Undergo Online Treatment Offered by the General Practitioner

Sociodemographic variables did not serve as significant predictors for willingness to undergo online treatment offered by the GP, but health-related information–seeking personality, social motive, existing experience with online communication with the GP, and willingness to undertake online communication with the GP significantly affected willingness to undergo online treatment offered by the GP. In terms of sociodemographic variables, the wealthier and more highly educated people were more willing to pay additionally for online treatment. Existing experience with online communication with a GP, willingness to undertake online communication with a GP, and willingness to undergo online treatment also significantly affected willingness to pay additionally for online treatment. The details of the multiple regression were gender, age, monthly household net income, and trust in the GP did not serve as significant predictors for willingness to undergo online treatment offered by the GP. PU had a positive influence on willingness to undergo online treatment offered by the GP (B=.092, *P*=.08), but the impact did not meet statistical significance. Furthermore, willingness to undergo online treatment offered by the GP could be predicted by the following variables on a 5% significance level, arranged in descending order: willingness to communicate online with the GP more often in the future (B=.495, *P*<.001), health-related information–seeking personality (B=.369, *P*<.001), actual use of online communication with the GP (B=.198, *P*<.001), and social motive (B=.178, *P*=.002). The adjusted *R* ^*2*^ was .546 (*F*_10,765_=94.191, *P*<.001), indicating that the dependent variable was explained quite well through the explanatory variables in the regression (Table 2).

**Table 2:** Explanatory variables to predict willingness to undergo online treatment offered by the GP.

Explanatory variables		B	*P*
Intercept		2.697	<.001
**Sociodemographic variables**			
	Gender	.025	.81
	Age	.004	.28
	Education	-.013	.78
	Monthly household net income	.008	.85
**Psychosocial variables**			
	Health-related information–seeking personality (factor score EFA1)	.369	<.001
	Social motive (factor score EFA2)	.178	.002
	Trust in the GP	-.061	.16
**Internet usage**			
	Actual use of online communication with the GP	.198	<.001
	Perceived usefulness of the Internet for health-related information (PU) (factor score EFA2)	.092	.08
	Willingness to communicate online with the GP more often in the future	.495	<.001

#### Multiple Regression Analysis to Predict Willingness to Pay Additionally for Online Treatment Offered by the General Practitioner

A second multiple regression was calculated with the same predictors as described previously and additionally with the variable “willingness to undergo online treatment offered by the GP” for the dependent variable “willingness to pay additionally for online treatment offered by the GP” (Table 3). With respect to sociodemographic variables, the wealthier and more highly educated people were more willing to pay additionally for online treatment. Existing experience with online communication with the GP, willingness to undertake online communication with the GP, and willingness to undergo online treatment also significantly affected willingness to pay additionally for online treatment offered by the GP. The details of this multiple regression analysis were gender, age, trust in the GP, and social motive did not serve as significant predictors. Health-related information–seeking personality (B=.127, *P*=.07) and PU (B=–.098, *P*=.09) both had a significant impact on willingness to pay additionally for online treatment offered by the GP, but these impacts did not meet statistical significance. The variables willingness to undergo online treatment offered by the GP (B=.391, *P*<.001), actual use of online communication with the GP (B=.192, *P*=.001), highest education level (B=.178, *P*<.001), monthly household net income category (B=.115, *P*=.01), and the willingness to communicate with the GP more often in the future (B=.076, *P*=.03) were significant predictors on a 5% level. The resulting adjusted *R* ^*2*^ was .361 (*F*_11,764_=39.308, *P*<.001).

**Table 3:** Explanatory variables to predict willingness to pay additionally for online treatment offered by GP.

Explanatory variables		B	*P*
Intercept		2.298	.62
**Sociodemographic variables**			
	Gender	–.013	.91
	Age	.001	.91
	Education	.178	<.001
	Monthly household net income	.115	.01
**Psychosocial variables**			
	Health-related information–seeking personality (factor score EFA1)	.127	.07
	Social motive (factor score EFA2)	.066	.30
	Trust in the GP	.032	.51
**Internet usage**			
	Actual use of online communication with the GP	.192	.001
	Perceived usefulness of the Internet for health-related information (PU) (factor score EFA2)	–.098	.09
	Willingness to communicate online with the GP more often in the future	.076	.03
	Willingness to undergo online treatment offered by the GP	.391	<.001

## Discussion

### Principal Findings

The sociodemographic variables age and gender and the psychosocial variable trust in the GP did not serve as significant predictors for either the willingness to undergo online treatment or the willingness to pay additionally for online treatment. Younger people were described as being more prone to switching to an online visit with a GP in another study [35], but the nonsignificant influence of age in our study deserves further consideration. One reason might be that participants in our study were selected via an online panel, so that participants (younger and older) in our sample are probably more open to online-related issues than the general public. Gender was not significant either. Hence, males and females did not differ in their general willingness to undergo online treatments and in their willingness to pay additionally for online treatments. More detailed analyses of other aspects of the online patient-physician relationship (eg, online correspondence, appointments) may allow additional insights into possible gender differences (eg, see Bidmon and Terlutter [66]). Trust in the GP also failed to be a significant predictor. This may be because patients do not perceive any differences in having to communicate with their GP face-to-face or online as long as they can contact their own GP, in whom they place their trust. Trust might play a more important role if online consultations are analyzed in which patient and physicians are less well-acquainted with each other (eg, if the patient contacts an unfamiliar online health care provider for advice). Other important findings of this study are that willingness to undergo online treatment is partly determined by the level of existing experience, willingness to communicate online with the GP, and health information-seeking personality and social motivation for such behavior. These findings are in line with Roger’s diffusion of innovation theory. This theory explains how and why new ideas and technologies are spread through different cultures. According to our results, early adopters are willing to undergo online treatments offered by the GP and pay for online treatment. Early adopters are characterized by a high social status and are more socially forward than late adopters, and they are characterized by higher available financial resources and a higher level of education. Early adopters are also opinion leaders for the other adopter categories, which implies that they may spread their opinion about and experiences with online treatments among others [67]. Furthermore, as mentioned previously, some patients inform themselves more extensively before visiting a physician and are more involved in the patient-physician interaction compared to others who do this to a lesser extent [53,55], which is in line with the interpretive and deliberative models of Emanuel and Emanuel [5]. This may imply that those people who are more involved in the patient-physician interaction (eg, the physician is responsible for explaining and interpreting the patient’s values, informing the patient, and implementing the patient’s selected interventions; the physician helps to reflect the patient’s preferences and values before making a decision) [5], have a higher need for information searching and are also more prone to looking online for health-related information, which can also satisfy their need for PU. Ascribing higher PU, which regards the Internet as a source for gaining health-related information easily, might lead to a higher adoption of undergoing an online treatment and paying extra for the online consultation [38]. Patients who already communicate with their GP online (eg, through email) might be more willing to undergo an online treatment and are more prone to pay for it [30]. Furthermore, it can be accentuated that people with a higher social motive (eg, people who use the Internet in order to be up-to-date, to establish contact with someone easily, to gather information anonymously, and/or people who like to share their knowledge with others) are more willing to try new technologies and new techniques, and are more willing to replace a face-to-face treatment with an online consultation.

The frequency distribution (see Multimedia Appendix 2) clearly shows that respondents are not willing to pay additionally for this service. Instead, they may even expect online treatments to be available at a lower price (ie, to be less expensive) [13,35]. The factor willingness to pay extra for online treatment is influenced additionally by the monthly household net income category and education level, which can be explained by the fact that more highly educated people usually have a higher monthly net income (ie, earn more) and may be more willing to pay for an online treatment.

### Limitations

There are some limitations within this study. The study was based on a patient online panel sample. Therefore, only patients with Internet access who visited a GP within the last 3 months before the survey were able to participate in the survey. As a consequence, participants of the survey may be more familiar with online health-related issues and are, therefore, more willing to undergo and pay for online treatments compared to patients without Internet access. However, the research question is especially relevant to those patients with some Internet affinity.

Furthermore, there are different legal backgrounds and restrictions in different European countries. For instance, online medical treatments offered by German physicians are not legally allowed at present [68,69]. Similar restricted regulations are found in Austria [70-73]. In other European countries, such as Switzerland or Great Britain, online medical treatments are already permitted and more established [24,74-76]. For this reason, we classify our study as exploratory in nature.

### Practical Implications and Directions of Future Research

In general, patients only show a medium willingness to undergo online treatments with a GP. However, 19.9% in our study indicated a very high willingness to undergo an online treatment, whereas another third (36.5%) clearly rejected the idea. These results of our study could be useful for the patients’ GPs and for online health care providers. GPs could offer online treatments to reduce waiting hours, to acquire new segments of patients, and to work in a manner that is more time-flexible; there is a viable portion of patients who would clearly appreciate such offers. Furthermore, GPs could offer predetermined time slots for in-person and online treatments and patients could be segmented according to their willingness to undergo an online treatment. This could be advantageous, especially for GPs who offer on-call services and have to be time-flexible and geographically independent. In a next step, the legal restrictions should be clarified and remuneration models should be discussed. Additionally, considerations must follow for which realms of physician-patient relationships are suitable for online treatments and which are not.

Furthermore, the results of this study could be useful for physicians who are considering offering online treatments for specified patient segments (eg, women or men, people with special chronic diseases, or employed people). If physicians know sociodemographic and psychosocial details about their patients who are willing to undergo online treatments, specific and time-flexible treatments may be tailored more easily. According to some surveys (eg, American Well [35]), younger people aged between 18 and 34 years, who may be more Internet literate and have a higher social motive, are more willing to undergo an online treatment. This may indicate that physicians should incorporate online treatments more into their practice and promote it through different online media channels (eg, social media channels such as Facebook or Twitter or on online physician-rating websites). In addition, online treatments should be affordable and are expected to cost less than personal face-to-face treatments.

The reputation of the GP associated with an online health care provider (eg, DoctorSpring.com [17]) might also have a significant influence on patient’s trust and on the patient’s willingness to use an online consultation with a physician whom the patient has not previously seen. Patients wish to select their physician on their own and want to know which medical school the GP has attended, their specialties, and their certifications. People will be more satisfied with an online treatment if they can see a picture of the physician, have the possibility to review the physician’s credentials, and can verify their board certification [35]. Therefore, to increase the patient’s trust in their physicians, GPs should satisfactorily show which school and additional educational programs they have attended by publishing verified certificates on the website. Moreover, physicians should offer patients the option to leave a review and/or a rating after an online consultation. One of the most important factors to ensure compliance is empathetic communication with the patient, while demonstrating competence [11]. Further studies should analyze the impact of online treatments on the information asymmetry between physicians and doctors, concordance, and compliance. Based on this study, results may also be useful for the improvement of online treatments by tailoring the websites of online health care providers to more accurately reflect the needs and requirements of the patients. The usability, accessibility, and design (eg, convenient handling, clear design, and easy access without an inconvenient log-in process) of the website [39,77,78] should match the patients’ needs to enhance the willingness to undergo an online treatment in the future. Furthermore, detailed information about the offered online treatments (eg, information about the different kinds of treatments, costs, and how to arrange an appointment) should be revealed on the website. When talking about the websites of online health care providers, the privacy of the patients should always be protected and this should be imparted in such a way that the users feel secure and enjoy undergoing an online treatment without fearing a lack of privacy. Although the legal and ethical aspects for offering and using online treatments are almost unknown in the public opinion, it would be interesting to ascertain how legal regulations influence the willingness to undergo online treatments and the willingness to pay additionally for online treatments. Future research should consider these important aspects. Other possible future research questions which arise based on the results of this study are if there are any differences between patients who are willing to undergo an online treatment and patients who are willing to pay for it, how the willingness to engage in online treatments can be influenced to match the requirements of the patients, or how the willingness to pay additionally for online treatments offered by the GP can be influenced to match the requirements of the patients. Other research topics could include issues such as which tariff model is appropriate for policymakers and what are the likely social and Internet usage factors that might shift the balance in favor of the willingness to undergo and pay for online treatments, how the requirements of patients who are not willing to undergo an online treatment and are not willing to pay for it could be matched, or how communication concepts appeal to patients. Last but not least, patients’ willingness to undergo online treatment and GPs’ willingness to offer online treatment may be conceptualized as a kind of concordance [79-82], referring to the usage of the Internet in the patient-physician relationship. This perceived concordance may lead to higher patient compliance and higher patient satisfaction similar to the results of studies dealing with age or gender concordance [80,83,84].

### Conclusion

Online treatments offer many opportunities for the health care sector and the future patient-physician relationship. Online treatments will certainly not replace face-to face treatments for acute or severe illnesses, for which a confirmed diagnosis is always mandatory, in the near future. As our study has demonstrated, willingness to undergo online treatment is limited and older people or people with complex health problems will probably avoid online treatments and prefer a face-to-face appointment with their GP [85,86]. There are also concerns regarding danger for those patients who use the Internet to search for health-related information (eg, misdiagnosis and exploitation) [87,88]. Online treatments will probably only be used by patients with common health issues (eg, headaches, sore throats, coughs, or chronic diseases) [85]. Telediagnosis using a combination of traditional face-to-face treatment and online treatment is on the rise [2] and will represent a strong future trend, which has already commenced now. Nevertheless, the physicians’ quality in the patient-physician relationship will remain the most important element, independent of the medium of communication [11].

## Appendix

Multimedia Appendix 1 Questions and justification of items (original language and translation for the paper).

Multimedia Appendix 2 Frequency distribution of willingness to undergo online treatment offered by the GP and willingness to pay additionally for online treatment offered by the GP.

## Abbreviations

EFA: exploratory factor analysis
GfK: Gesellschaft für Konsumforschung
GP: general practitioner
PEOU: perceived ease-of-use
PU: perceived usefulness
