# Supplementary material for: What Predicts Patients’ Willingness to Undergo Online Treatment and Pay for Online Treatment? Results from a Web-Based Survey to Investigate the Changing Patient-Physician Relationship
Source: J Med Internet Res. 2016 Feb 4;18(2):e32. doi: 10.2196/jmir.5244 (PMC4782912; doi:10.2196/jmir.5244)
Supplement: Multimedia Appendix 1 [file jmir_v18i2e32_app1.pdf]

# MULTIMEDIA APPENDIX 1 - Questions and Justification of Items (Original Language and Translation for the Paper)

## SOZIALES MOTIV & PU / SOCIAL MOTIVE & PU

1. **Bitte kreuzen Sie an, aus welchen Gründen Sie das Internet für die Suche nach gesundheitsorientierten Informationen nutzen?**

| | Ich nutze das Internet, weil … | Trifft überhaupt nicht zu | | | | | | Trifft vollkommen zu | Keine Antwort |
|---|---|---|---|---|---|---|---|---|---|
| F11_1 | … ich auf einfache Art und Weise Recherchen machen kann. | 1 | 2 | 3 | 4 | 5 | 6 | 7 | 8 |
| F11_2 | … es die Informationssuche für mich erleichtert. | 1 | 2 | 3 | 4 | 5 | 6 | 7 | 8 |
| F11_3 | … es meine Produktivität bei der Suche nach gesundheitsorientierten Informationen steigert. | 1 | 2 | 3 | 4 | 5 | 6 | 7 | 8 |
| F11_4 | … ich schnell gelernt habe damit umzugehen. | 1 | 2 | 3 | 4 | 5 | 6 | 7 | 8 |
| F11_5 | … es mir eine Vielzahl an Informationen bietet. | 1 | 2 | 3 | 4 | 5 | 6 | 7 | 8 |
| F11_6 | … die Informationen aktuell sind. | 1 | 2 | 3 | 4 | 5 | 6 | 7 | 8 |
| F11_7 | … die Informationen einfach zu verstehen sind. | 1 | 2 | 3 | 4 | 5 | 6 | 7 | 8 |
| F11_8 | … es mir richtige Informationen bietet. | 1 | 2 | 3 | 4 | 5 | 6 | 7 | 8 |
| F11_9 | ... es mir unterschiedliche Formate, wie z.B. Social Networks, Podcasts oder Gesundheitsforen bietet. | 1 | 2 | 3 | 4 | 5 | 6 | 7 | 8 |
| F11_10 | … ich Zeit sparen möchte. | 1 | 2 | 3 | 4 | 5 | 6 | 7 | 8 |
| F11_11 | … ich auf einfache Art und Weise mit jemandem in Kontakt treten kann. | 1 | 2 | 3 | 4 | 5 | 6 | 7 | 8 |
| F11_12 | … ich am Puls der Zeit sein möchte. | 1 | 2 | 3 | 4 | 5 | 6 | 7 | 8 |
| F11_13 | … ich mich lieber anonym informieren möchte. | 1 | 2 | 3 | 4 | 5 | 6 | 7 | 8 |
| F11_14 | … eine Nutzung 24 Stunden 7 Tage möglich ist. | 1 | 2 | 3 | 4 | 5 | 6 | 7 | 8 |
| F11_15 | … ich mein Wissen mit anderen teilen kann. | 1 | 2 | 3 | 4 | 5 | 6 | 7 | 8 |
| F11_16 | … dadurch der Sucherfolg von Informationen gesteigert wird. | 1 | 2 | 3 | 4 | 5 | 6 | 7 | 8 |
| F11_17 | … es Spaß macht, es zu nutzen. | 1 | 2 | 3 | 4 | 5 | 6 | 7 | 8 |
| F11_18 | … ich finde, dass es unterhaltsam ist. | 1 | 2 | 3 | 4 | 5 | 6 | 7 | 8 |

**Why do you use the Internet for health-related information searches? Please mark all answers that apply.**

| | I use the Internet because … | Strongly disagree | | | | | | Strongly agree | No answer | Adapted from |
|---|---|---|---|---|---|---|---|---|---|---|
| F11_1 | … I can search online easily. | 1 | 2 | 3 | 4 | 5 | 6 | 7 | 8 | [7,59] |
| F11_2 | … it simplifies the information search for me. | 1 | 2 | 3 | 4 | 5 | 6 | 7 | 8 | [40,44,45,47] |
| F11_3 | … it enhances my productivity in health-related information searching. | 1 | 2 | 3 | 4 | 5 | 6 | 7 | 8 | [40,44,45,47] |
| F11_4 | … I have learnt quickly how to handle it. | 1 | 2 | 3 | 4 | 5 | 6 | 7 | 8 | [40,44,47] |
| F11_5 | … it offers a variety of information. | 1 | 2 | 3 | 4 | 5 | 6 | 7 | 8 | [7,59] |
| F11_6 | … information is up-to-date. | 1 | 2 | 3 | 4 | 5 | 6 | 7 | 8 | [7,61] |
| F11_7 | … information can be understood easily. | 1 | 2 | 3 | 4 | 5 | 6 | 7 | 8 | [7,59] |
| F11_8 | … it offers the right information. | 1 | 2 | 3 | 4 | 5 | 6 | 7 | 8 | [40,47] |
| F11_9 | ... it offers different formats, like e.g. social networks, podcasts or health fora. | 1 | 2 | 3 | 4 | 5 | 6 | 7 | 8 | [7,59,60] |
| F11_10 | … I want to save time. | 1 | 2 | 3 | 4 | 5 | 6 | 7 | 8 | [7] |
| F11_11 | … I can establish contact with someone easily. | 1 | 2 | 3 | 4 | 5 | 6 | 7 | 8 | [7,61] |
| F11_12 | … I want to be up-to-date. | 1 | 2 | 3 | 4 | 5 | 6 | 7 | 8 | [7] |
| F11_13 | … I prefer to gather information anonymously. | 1 | 2 | 3 | 4 | 5 | 6 | 7 | 8 | [7,60] |
| F11_14 | … usage is possible for 24 hours on 7 days. | 1 | 2 | 3 | 4 | 5 | 6 | 7 | 8 | [7,60] |
| F11_15 | … I can share my know-how with others. | 1 | 2 | 3 | 4 | 5 | 6 | 7 | 8 | [7] |
| F11_16 | … the success of finding information can be increased. | 1 | 2 | 3 | 4 | 5 | 6 | 7 | 8 | [40,44,47] |
| F11_17 | … it is fun to use. | 1 | 2 | 3 | 4 | 5 | 6 | 7 | 8 | [44,62,63] |
| F11_18 | … I find it entertaining. | 1 | 2 | 3 | 4 | 5 | 6 | 7 | 8 | [44,63,64] |

## INTERNETKOMMUNIKATION MIT DEM ARZT / INTERNET COMMUNICATION WITH THE PHYSICIAN

**F13** **Im Folgenden werden Fragen zum Thema Internetkommunikation mit Ihrem Allgemeinarzt/Ihrer Allgemeinärztin behandelt. Bitte kreuzen Sie an, wie häufig Sie das Internet für die Kommunikation mit Ihrem Allgemeinarzt/Ihrer Allgemeinärztin nutzen.**

| Wie häufig nutzen Sie das Internet, um … | Täglich | Wöchentlich | Seltener als wöchentlich | Monatlich | Seltener als monatlich | Nie |
|---|---|---|---|---|---|---|
| … mit Ihrer/m Allgemeinärztin/Allgemeinarzt zu kommunizieren? | 1 | 2 | 3 | 4 | 5 | 6 |

**F13** **The following question relates to the topic of Internet communication with your general practitioner (GP). Please indicate how often you use the Internet for the communication with your GP at present.**

| How often do you use the Internet to … | daily | weekly | less often than weekly | monthly | less often than monthly | never |
|---|---|---|---|---|---|---|
| … communicate with your general practitioner (GP)? | 1 | 2 | 3 | 4 | 5 | 6 |

Edited by the research team

**F15** **Können Sie sich vorstellen, in der Zukunft, häufiger mit Ihrem Allgemeinarzt/Ihrer Allgemeinärztin über das Internet zu kommunizieren?**

| Kann ich mir überhaupt nicht vorstellen | | | | | | Kann ich mir sehr gut vorstellen | Keine Antwort |
|---|---|---|---|---|---|---|---|
| 1 | 2 | 3 | 4 | 5 | 6 | 7 | 8 |

**F15** **Can you imagine using the Internet more often in the future for communicating with your GP?**

| Highly unlikely | | | | | | Very likely | No answer |
|---|---|---|---|---|---|---|---|
| 1 | 2 | 3 | 4 | 5 | 6 | 7 | 8 |

Edited by the research team

**F18** **Wie wichtig ist es Ihnen, auch eine Online-Behandlung nutzen zu können?**

| Ist mir überhaupt nicht wichtig. | | | | | | Ist mir sehr wichtig. | Keine Antwort |
|---|---|---|---|---|---|---|---|
| 1 | 2 | 3 | 4 | 5 | 6 | 7 | 8 |

**F18** **How important is it for you to be able to undergo an online treatment by the GP?**

| Not important at all. | | | | | | Very important. | No answer |
|---|---|---|---|---|---|---|---|
| 1 | 2 | 3 | 4 | 5 | 6 | 7 | 8 |

Edited by the research team

**F19** **Inwiefern wären Sie bereit für eine Online-Behandlung einen gewissen Betrag zuzuzahlen?**

| Ich wäre dazu überhaupt nicht bereit | | | | | | Ich wäre dazu bereit | Keine Antwort |
|---|---|---|---|---|---|---|---|
| 1 | 2 | 3 | 4 | 5 | 6 | 7 | 8 |

**F19** **Indicate how willing you would be to pay a certain amount additionally for online treatment?**

Edited by the research team

| I would not be willing at all | | | | | | I would be willing | No answer |
|---|---|---|---|---|---|---|---|
| 1 | 2 | 3 | 4 | 5 | 6 | 7 | 8 |

## HEALTH-RELATED INFORMATION SEEKING PERSONALITY

**Bitte geben Sie im Folgenden an, wie sehr die einzelnen Aussagen auf Sie zutreffen.**

| | | Trifft über-haupt nicht zu | | | | | | Trifft vollkommen zu | Keine Antwort |
|---|---|---|---|---|---|---|---|---|---|
| F20_1 | Es ist wichtig, einem Arzt bzw. einer Ärztin bereits gut informiert gegenüberzutreten. | 1 | 2 | 3 | 4 | 5 | 6 | 7 | 8 |
| F20_2 | Wenn ich mich über Krankheiten im Internet informiere, habe ich das Bedürfnis, mit meinem Arzt bzw. meiner Ärztin darüber zu sprechen. | 1 | 2 | 3 | 4 | 5 | 6 | 7 | 8 |
| F20_3 | Wenn ich eine Therapie verschrieben bekomme, erkundige ich mich nach Alternativtherapien im Internet. | 1 | 2 | 3 | 4 | 5 | 6 | 7 | 8 |
| F20_4 | Manchmal habe ich das Gefühl, besser über meinen Zustand informiert zu sein, als mein Arzt bzw. meine Ärztin. | 1 | 2 | 3 | 4 | 5 | 6 | 7 | 8 |
| F20_5 | Ist der Patient/die Patientin informiert, wird die Kommunikation mit dem Arzt/der Ärztin dadurch verbessert. | 1 | 2 | 3 | 4 | 5 | 6 | 7 | 8 |
| F20_6 | Erst nach einer Recherche im Internet entscheide ich, ob ein Arztbesuch notwendig ist. | 1 | 2 | 3 | 4 | 5 | 6 | 7 | 8 |
| F20_7 | Wenn mir Medikamente verschrieben wurden, suche ich Informationen darüber im Internet. | 1 | 2 | 3 | 4 | 5 | 6 | 7 | 8 |
| F20_8 | Ist der Patient/die Patientin informiert, nimmt sich der Arzt/die Ärztin mehr Zeit für die Behandlung. | 1 | 2 | 3 | 4 | 5 | 6 | 7 | 8 |
| F20_9 | Der Arzt/die Ärztin verschreibt eher ein gewünschtes Medikament, wenn der Patient/die Patientin informiert ist. | 1 | 2 | 3 | 4 | 5 | 6 | 7 | 8 |

**Please indicate how well the following statements apply to you.**

| | | Strongly disagree | | | | | | Strongly agree | No answer | |
|---|---|---|---|---|---|---|---|---|---|---|
| F20_1 | It is important to me to be well-informed when consulting a physician. | 1 | 2 | 3 | 4 | 5 | 6 | 7 | 8 | Adapted from [56,57] |
| F20_2 | When I obtain health information from the Internet, I need to talk about this information with my physician. | 1 | 2 | 3 | 4 | 5 | 6 | 7 | 8 | Edited by the research team |
| F20_3 | When a therapy is prescribed for me, I look for alternative therapies on the Internet. | 1 | 2 | 3 | 4 | 5 | 6 | 7 | 8 | Adapted from [57,58] |
| F20_4 | Sometimes I have the feeling that I am better informed about my medical condition than my physician. | 1 | 2 | 3 | 4 | 5 | 6 | 7 | 8 | Edited by the research team |
| F20_5 | If the patient is informed, the communication with the physician is improved. | 1 | 2 | 3 | 4 | 5 | 6 | 7 | 8 | |
| F20_6 | I only decide whether a consultation with a physician is really necessary, once I have conducted some health information searches on the Internet. | 1 | 2 | 3 | 4 | 5 | 6 | 7 | 8 | |
| F20_7 | If some medicines have been prescribed, I look for information about them on the Internet. | 1 | 2 | 3 | 4 | 5 | 6 | 7 | 8 | Adapted from [57,58] |
| F20_8 | If the patient is informed, the physician allows more time for the treatment. | 1 | 2 | 3 | 4 | 5 | 6 | 7 | 8 | Edited by the research team |
| F20_9 | The physician is more likely to prescribe a requested medicine, if the patient is informed. | 1 | 2 | 3 | 4 | 5 | 6 | 7 | 8 | |

## EINSTELLUNG ZUM ARZT/ARZTDATEN/ARZTKRITERIEN SOLL-IST-ZUSTAND / ATTITUDE TOWARD THE PHYSICIAN/PHYSICIAN DATA/PHYSICIAN CRITERIA NOMINAL-ACTUAL COMPARISON

F34 **Wie groß ist Ihr Vertrauen zu Ihrem Allgemeinarzt / Ihrer Allgemeinärztin?**

| Überhaupt kein Vertrauen | | | | | | Sehr großes Vertrauen | Keine Antwort |
|---|---|---|---|---|---|---|---|
| 1 | 2 | 3 | 4 | 5 | 6 | 7 | 8 |

F34 **How much do you trust your GP?**

| No trust at all | | | | | | Very high trust | No answer |
|---|---|---|---|---|---|---|---|
| 1 | 2 | 3 | 4 | 5 | 6 | 7 | 8 |

Edited by the research team

## DEMOGRAPHISCHE ANGABEN / SOCIODEMOGRAPHIC DATA

D1 **Geschlecht:** [1] männlich
[2] weiblich

D2_1 **Geburtsjahr:** _____________

D1 **Gender:** [1] male
[2] female

D2_1 **Year of birth:** _____________

**Höchste abgeschlossene Ausbildung:** (D4)

1 Schüler in allgemeinbildender Schule (ohne Schulabschluss)
2 Haupt-/Volksschulabschluss ohne Lehre
3 Haupt-/Volksschulabschluss mit Lehre
4 Weiterführende Schule ohne Abitur (Realschulabschluss / Mittlere Reife)
5 Abitur / (Fach-) Hochschulreife ohne Studium
6 Abitur / (Fach-) Hochschulreife mit Studium
7 Promotion/ Habilitation
8 Keine Antwort

**Highest level of education:** (D4)

1 Without school qualification
2 Secondary general school
3 Polytechnic secondary school
4 Intermediate secondary school
5 High school diploma / A-levels
6 University degree
7 Postdoctoral degree / Professor
8 No answer

**Monatliches Nettohaushaltseinkommen:** (D8)

1 < 1.500 € 2 1.500–2.500 3 2.501–3.500 4 3.501–4.500 5 > 4.500 6 Keine Antwort

**Monthly household net income:** **(D8)**

**1** < 1.500 € **2** 1.500–2.500 **3** 2.501–3.500 **4** 3.501–4.500 **5** > 4.500 **6** No answer
