# Supplementary material for: What Predicts Patients’ Willingness to Undergo Online Treatment and Pay for Online Treatment? Results from a Web-Based Survey to Investigate the Changing Patient-Physician Relationship
Source: J Med Internet Res. 2016 Feb 4;18(2):e32. doi: 10.2196/jmir.5244 (PMC4782912; doi:10.2196/jmir.5244)

MULTIMEDIA APPENDIX 2 – Frequency Distribution of Willingness to Undergo Online Treatment Offered by the GP and Willingness to Pay Additionally for Online Treatment Offered by the GP

The bars show the frequency distribution of number of answers for willingness to be able to undergo online treatment offered by the GP (F18) and willingness to pay additionally for online treatment offered by the GP (F19).

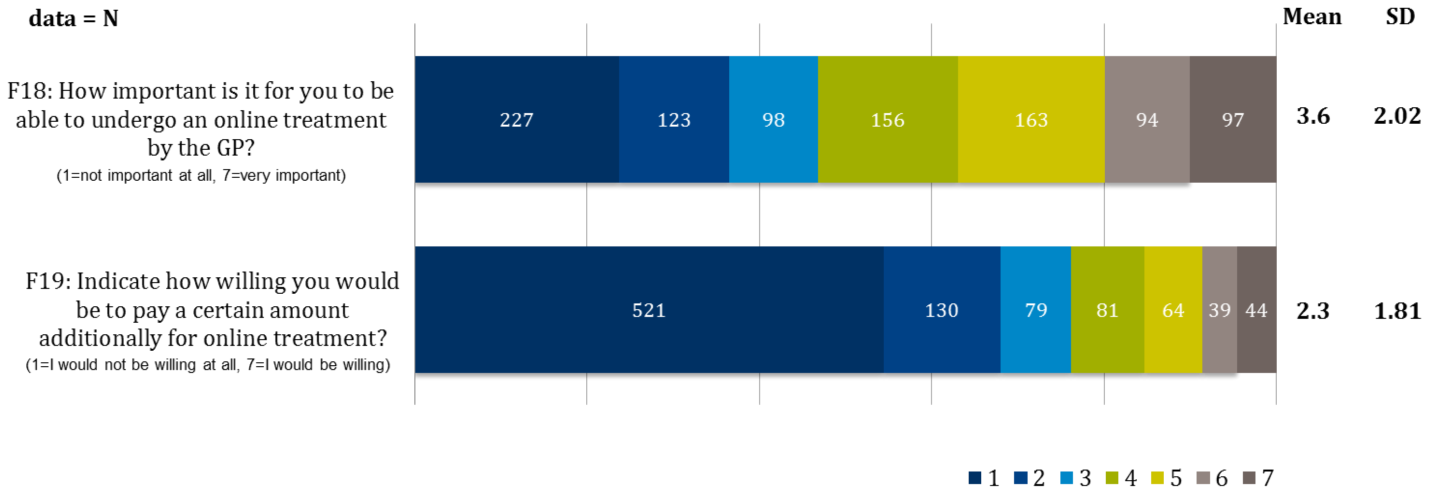

Supplement: Multimedia Appendix 2 [file jmir_v18i2e32_app2.pdf]
